# PReS-FINAL-2280: Lupus disease activity in a pediatric Colombian cohort with systemic lupus erytematosus

**DOI:** 10.1186/1546-0096-11-S2-P270

**Published:** 2013-12-05

**Authors:** AS Diaz Maldonado, A Monje Gaitan, N Gamba, F Gonzalez

**Affiliations:** 1 Reumatologia Pediatrica, Hospital de La Misericordia, Bogota, Colombia

## Introduction

Systemic Lupus Erythematosus (SLE) is disease which may have severe and variable activity in the pediatric population.

## Objectives

compare the index of activity in two times at cohort of pediatric patients with SLE.

## Methods

Analytic descriptive study. 89 patients with diagnosis SLE (1986 ACR criteria) from a rheumatology center at a pediatric hospital were evaluated at the time of diagnosis and twelve months after. Medical records were reviewed registering the following variables: score SLEDAI, Antinuclear antibodies (ANAs), Anti-DNA antibodies and complement C3 and C4 levels. Analysis was done through parametric and non parametric tests to compare means and proportions using STATA11. Shapiro-Wilk and Wilcoxon tests (non-normally distributed data) were applied.

## Results

Median score SLEDAI at the time of diagnosis was 23 (min 4 max 61); after 12 months it was 4 (min 0 max 31) (p = 0.001). Antinuclear antibodies (ANAs) reactivity at the diagnosis was 88,1%; after 12 months it was 6,67% (p = 0.17). Anti-DNA antibodies reactivity at the diagnosis was 66,7%; after 12 months it was 56,41% (p = 0.01). C3 level was diminished in 67,95% of patients at the diagnosis; after 12 months it was diminished in 33,3% of patients (p = 0.01). C4 level was diminished in 71,7% of patients at the diagnosis; after 12 months it was diminished in 40% of patients (p = 0.01).

## Conclusion

Statistical significance was documented regarding score SLEDAI, Anti-DNA antibodies reactivity, hypocomplementemia for the time of diagnosis and twelve month follow-up. This suggests that improvement in disease activity was due to the established treatment.

## Disclosure of interest

None declared.

